# Rapid Simultaneous Testing of Multiple Antibiotics by the MALDI-TOF MS Direct-on-Target Microdroplet Growth Assay †

**DOI:** 10.3390/diagnostics11101803

**Published:** 2021-09-29

**Authors:** Evgeny A. Idelevich, Ilka D. Nix, Janika A. Busch, Katrin Sparbier, Oliver Drews, Markus Kostrzewa, Karsten Becker

**Affiliations:** 1 Friedrich Loeffler-Institute of Medical Microbiology, University Medicine Greifswald, 17475 Greifswald, Germany; evgeny.idelevich@med.uni-greifswald.de; 2 Institute of Medical Microbiology, University Hospital Münster, 48149 Münster, Germany; ilka.nix@bruker.com (I.D.N.); alex.busch95@gmx.de (J.A.B.); 3 Bruker Daltonics GmbH & Co. KG, 28359 Bremen, Germany; katrin.sparbier@bruker.com (K.S.); oliver.drews@bruker.com (O.D.); markus.kostrzewa@bruker.com (M.K.)

**Keywords:** MALDI-TOF, direct-on-target microdroplet growth assay, rapid susceptibility testing, multiple antibiotics, multiplex testing, *Enterobacterales*

## Abstract

Accelerating antimicrobial susceptibility testing (AST) is a priority in the development of novel microbiological methods. The MALDI-TOF MS-based direct-on-target microdroplet growth assay (DOT-MGA) has recently been described as a rapid phenotypic AST method. In this proof-of-principle study, we expanded this method to simultaneously test 24 antimicrobials. An *Enterobacterales* panel was designed and evaluated using 24 clinical isolates. Either one or two (only for antimicrobials with the EUCAST “I” category) breakpoint concentrations were tested. Microdroplets containing bacterial suspensions with antimicrobials and growth controls were incubated directly on the spots of a disposable MALDI target inside a humidity chamber for 6, 8 or 18 h. Broth microdilution was used as the standard method. After 6 and 8 h of incubation, the testing was valid (i.e., growth control was successfully detected) for all isolates and the overall categorical agreement was 92.0% and 92.7%, respectively. Although the overall assay performance applying short incubation times is promising, the lower performance with some antimicrobials and when using the standard incubation time of 18 h indicates the need for thorough standardization of assay conditions. While using “homebrew” utensils and provisional evaluation algorithms here, technical solutions such as dedicated incubation chambers, tools for broth removal and improved software analyses are needed.

## 1. Introduction

Rapid microbiological diagnostics are of considerable importance, as they contribute to the optimization of patient management [1] and improve clinical outcome [2,3]. With the introduction of matrix-assisted laser desorption/ionization time-of-flight mass spectrometry (MALDI-TOF MS) into the diagnostic routine, considerable acceleration of microbial identification has been achieved [4,5]. However, such progress is lacking in routine antimicrobial susceptibility testing (AST), which is at least as important for therapeutic decisions as identification [6]. The AST findings are usually only available on the next day after test initiation [7].

In recent years, several techniques have been attempted to determine microbial resistance by MALDI-TOF MS [8,9]. Indeed, combining identification and AST on a single platform could result in synergistic effects for workflow and cost saving [6]. However, the ultimate goal is to develop a phenotypic AST assay that is universal and independent from resistance mechanisms [10,11]. The AST results should be available within a working shift to enable prompt adjustment of antimicrobial treatment on the same day [6].

The MALDI-TOF MS-based direct-on-target microdroplet growth assay (DOT-MGA) has recently been suggested as a rapid universal phenotypic AST method [11,12,13,14]. Initially, Idelevich et al. [12] introduced the DOT-MGA to determine carbapenem susceptibility in *Klebsiella pneumoniae* and *Pseudomonas aeruginosa* while microdroplets of bacterial suspension with and without the addition of antibiotics were incubated on a surface of a MALDI target and, after a short incubation time, growth or no growth of bacteria were detected by MALDI-TOF MS measurement to determine susceptibility or resistance. Further applications followed for various antibiotics and bacterial species [11,12,13,14].

In this study, we expanded this method to the proof-of-principle simultaneous testing of multiple antibiotics at breakpoint concentrations. For this purpose, we designed and evaluated DOT-MGA antibiotic panels for *Enterobacterales*, a clinically very important bacterial group in which antibiotic resistance is increasing worldwide. The panel included the most clinically relevant antimicrobials with EUCAST breakpoints available for these organisms.

## 2. Materials and Methods

### 2.1. Bacterial Strains

The strain collection of *Enterobacterales* consisted of twelve prospectively and consecutively collected clinical isolates, as well as twelve consecutive clinical multidrug resistant (MDR) isolates. In total, 24 *Enterobacterales* isolates comprised *Klebsiella pneumoniae* (*n* = 8), *Escherichia coli* (*n* = 4), *Enterobacter cloacae* complex (*n* = 4), *Klebsiella oxytoca* (*n* = 2), *Klebsiella aerogenes* (*n* = 2), *Citrobacter koseri* (*n* = 2), *Citrobacter freundii* (*n* = 1) and *Serratia marcescens* (*n* = 1). All isolates originated from routine diagnostics of the Institute of Medical Microbiology, University Hospital Münster, Münster, Germany. Only one isolate per patient was included. Microorganisms were isolated from urine (*n* = 7), tracheal fluid (*n* = 3), superficial swab (*n* = 3), deep swab (*n* = 2), tissue (*n* = 2), implant (*n* = 2), feces (*n* = 2), sputum (*n* = 1), blood culture (*n* = 1) and bronchial lavage (*n* = 1).

### 2.2. Antimicrobials

Antibiotic panels were composed to include 24 antimicrobials (Table 1 and Table S1) for which EUCAST breakpoints (version 9.0) were available for *Enterobacterales* [15]. To allow complete categorization as susceptible, standard dosing regimen (S), susceptible, increased exposure (I) or resistant (R) according to EUCAST breakpoints [15], one concentration was tested for antimicrobials for which no I category exists, and two concentrations were tested for antimicrobials with the I category available from EUCAST [15]. For that, antibiotics contained in the respective wells of a Micronaut-S microtiter plate (MERLIN Diagnostika, Bornheim-Hersel, Germany) were used as described below.

### 2.3. MALDI-TOF MS Direct-on-Target Microdroplet Growth Assay (DOT-MGA)

**Figure 1:**
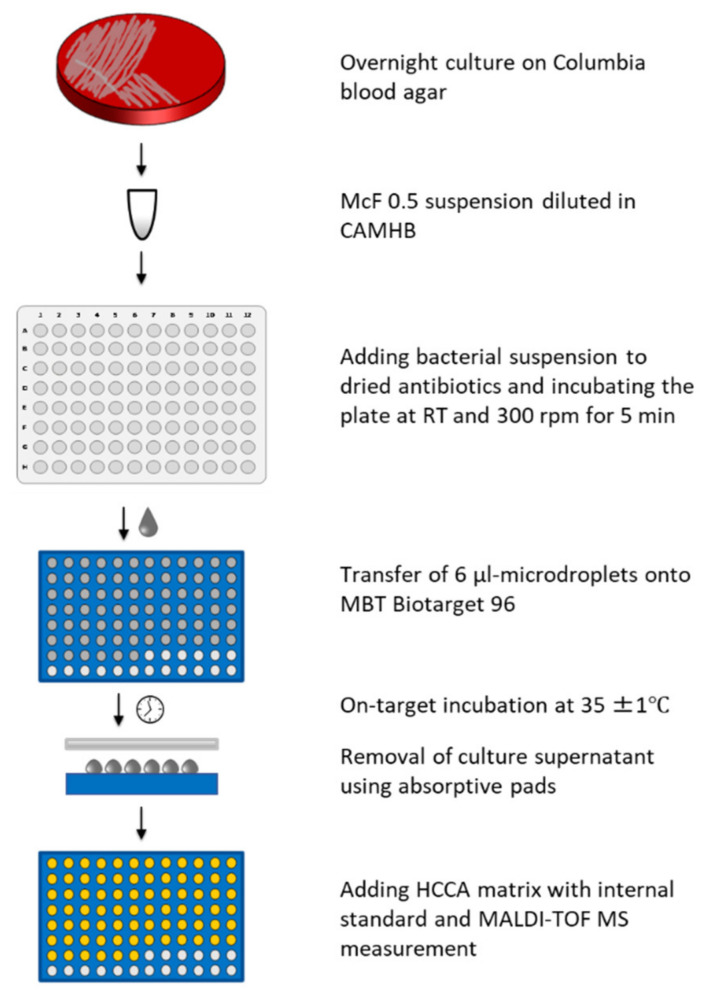
Workflow of the MALDI-TOF MS direct-on-target microdroplet growth assay (DOT-MGA).

Bacterial suspensions with turbidity of 0.5 McFarland standard were prepared in 0.9% saline from cultures incubated overnight on Columbia blood agar. These suspensions were diluted 1:200 in cation-adjusted Mueller-Hinton broth (CAMHB, BD Diagnostic, Heidelberg, Germany) to produce inoculum of approximately 5 × 10^5^ cfu/mL. Final inoculum size was confirmed by plating onto tryptic soy agar plates in triplicate and colony counting after overnight incubation (18–24 h). Several 100-µL samples of prepared inoculum were added into the wells of a Micronaut-S microtiter plate containing dried antibiotics (MERLIN Diagnostika, Bornheim-Hersel, Germany), followed by shaking the plate for 5 min at 300 rpm at room temperature to ensure complete dissolution of antibiotics. Several 6-µL samples of bacterial suspensions with antibiotics, as well as a growth control without antibiotic, were spotted in duplicate from the wells of a microtiter plate onto the spots of disposable MALDI targets (MBT Biotarget 96, Bruker Daltonics GmbH & Co. KG, Bremen, Germany) as microdroplets. For each isolate, three separate targets were prepared to test three different incubation times. The inoculated targets were incubated at 36°C for 6, 8 or 18 h in a plastic box (Bruker Daltonics GmbH & Co. KG, Bremen, Germany) containing 4 mL water to avoid evaporation of microdroplets. After incubation, medium was removed by absorptive pads, as previously described [12,16,17,18,19], but introducing the modification that the absorptive pad is applied directly from the top of microdroplets to remove broth from all the spots on a target in a single action (Figure 1). An amount of 1 μL MBT FAST Matrix (Bruker Daltonics GmbH & Co. KG, Bremen, Germany) containing an internal standard was added to each spot, and MALDI-TOF MS measurement performed twice for each spot with a MALDI Biotyper smart instrument (Bruker Daltonics GmbH & Co. KG, Bremen, Germany) and the DOT-MGA settings as previously described [19].

Mass spectra were interpreted using the MBT FAST prototype software (Bruker Daltonics GmbH & Co. KG, Bremen, Germany). In case of bacterial growth in a microdroplet with antibiotic, bacterial biomass is successfully detected by MALDI-TOF MS after the incubation period, and the result is interpreted as bacterial resistance against the tested concentration of an antimicrobial. In contrast, failed detection of bacterial biomass by MALDI-TOF MS defines inhibition of bacterial growth by an antibiotic in the tested concentration. The categorization of isolates as S, I or R was performed based on results for one or two breakpoint concentrations included for each antibiotic. The test was defined as valid, if the bacterial growth in control without an antibiotic was successfully detected by the MBT FAST prototype software.

The rates of categorical agreement (CA), very major error (VME, the number of false S results with DOT-MGA divided by the number of R results with standard method), major error (ME, the number of false R results with DOT-MGA divided by the number of S results with standard method) and minor error (mE, the number of false categorizations involving I category) were calculated for each antibiotic and each time point separately, as recommended by ISO [20] and FDA [21].

### 2.4. Broth Microdilution as Standard Method

After 6-µL samples were removed for DOT-MGA from the microtiter plates (MERLIN Diagnostika, Bornheim-Hersel, Germany) containing bacterial suspension and antibiotics, the incubation of these microtiter plates was performed for 18 ± 2 h at 35 ± 1 °C, as recommended by ISO [22] and CLSI [23] for broth microdilution method. The turbidity reading was performed visually, and the categorization results from the microtiter plate that was used for inoculation of the 18 h-DOT-MGA setup were used as reference for accuracy evaluation of the DOT-MGA.

Reference strains: *E. coli* ATCC 25922, *E. coli* ATCC 35218, *K. pneumoniae* ATCC 700603, *K. pneumoniae* ATCC BAA-1705 and *Pseudomonas aeruginosa* ATCC 27853 were used as quality control (QC).

## 3. Results

To evaluate the assay performance and compare it with the reference method, overall validity, categorical agreements (CA), very major errors (VME), major errors (ME) and minor errors (mE) were calculated for each antibiotic and each time point separately.

When the short incubation time of 6 h was implemented, the testing was valid (growth control detected) for all isolates and the overall CA was 92.0% (Table 1). With this incubation time, CA for individual antimicrobials varied between 70.8% (for cefepime) and 100% (for cefuroxime, cefotaxime, ceftazidime/avibactam, ertapenem, ciprofloxacin, levofloxacin, gentamicin, tobramycin and tigecycline). CA of ≥90% was achieved for 18 of 24 antimicrobials using this incubation duration.

After the 8-h incubation, all tests were valid, and the overall CA amounted to 92.7%. Applying this incubation duration, CA for individual antimicrobials ranged from 75.0% (for cefepime) to 100% (for cefuroxime, cefotaxime, ceftazidime/avibactam, ciprofloxacin, levofloxacin, moxifloxacin, gentamicin, tobramycin, trimethoprim/sulfamethoxazole and tigecycline). CA of ≥90% was achieved for 17 of 24 antimicrobials with the incubation for 8 h (Table 1).

Using the standard incubation time of 18 h, the test was valid (successful detection of growth control) in 75.2% of isolates and the CA calculated for valid tests was 90.5%. CA for individual antimicrobials varied between 77.8% (for ampicillin, cefepime and imipenem) and 100% (for cefotaxime, ceftazidime/avibactam, moxifloxacin, gentamicin, tobramycin and tigecycline). CA of ≥90% was achieved for 12 of 24 antimicrobials with the incubation for 18 h (Table 1).

## 4. Discussion

DOT-MGA is a novel MALDI-TOF MS-based AST method, which was reported to provide information on antimicrobial susceptibility or resistance in shorter time than the reference AST methods [11,12,13,14,16,17,18,19]. The assay principally relies on broth microdilution and is, therefore, a phenotypic growth-based method. Hence, potentially every antimicrobial can be tested with this universal method. Nevertheless, specific differences have been implemented in DOT-MGA, compared to the reference broth microdilution method. While the accuracy of the DOT-MGA has previously been demonstrated for several antimicrobials [12,16,17,18,19], the deviations from the standard method may hypothetically cause specific result deviation for particular antimicrobials not previously investigated, as it may be the case with every modified method. Such unknown effects may theoretically be caused, e.g., by the interference of antimicrobial substances with MALDI-TOF MS measurement, behavior of microorganisms in the presence of a specific antibiotic when incubated in microdroplets instead of 100-µL volume in a microtiter plate or microbial behavior on the surface of a MALDI target plate in the presence of a specific antibiotic. Therefore, this study aimed to test a wide range of antimicrobials from different classes in order to reveal any potential risk of inaccuracies with specific antimicrobials.

The short incubation time of 6 h resulted in an overall CA of 92.0% (Table 1), and the CA amounted to 92.7% after 8 h of incubation time. The overall CA after standard incubation time of 18 h was 90.5%. The individual performance of each antibiotic varied between 70.8% (for cefepime after 6 h incubation time) and 100% (for a large number of antibiotics after respective incubation time).

While the tested collection of consecutive clinical isolates reflected the occurrence in diagnostics routine, the number of resistant or susceptible strains was low for some antibiotics. Taking into account that the rates of VME and ME are calculated using the number of resistant or susceptible isolates, respectively, as denominator [20,21], the informative value of these parameters should be interpreted with caution (Table 1).

The best test performance was achieved when the short incubation times of six and eight hours were applied. The lower assay performance with the standard incubation time of 18 h is probably due to the fact that assay conditions and the evaluation algorithms were developed for a rapid test. With longer incubation, evaporation in microdroplets may become an issue that disturbs the microbial growth and the MALDI-TOF MS measurement. Indeed, a better control of humidity can be achieved with a dedicated incubation chamber than with the simple plastic box used in this study. Generally, this study was performed using “homebrew” utensils and a provisional software prototype.

Certainly, better control over the test conditions and, hence, better accuracy can be reached with standardized assay tools. The number of samples tested should also be increased and a broader range of resistance patterns tested to provide more reliable information on test performance. For the future, automated processing, standardized incubation tools and improved software analysis are needed that would enable comfortable workflow and increased test performance.

The MALDI-TOF MS-based DOT-MGA was shown in this proof-of-principle study to be a universal AST method suitable for rapid simultaneous testing of multiple antibiotics against clinical *Enterobacterales* isolates.

## Figures and Tables

**Table 1:** Results of simultaneous DOT-MGA testing of multiple antibiotics vs. *Enterobacterales*, *n* = 24.

Antibiotics	Isolate-Antibiotic Combinations, n ^a^			6 h Incubation					8 h Incubation					18 h Incubation				
				Valid Tests, %	Errors, n (%) ^b^			CA, %	Valid Tests, %	Errors, n (%) ^b^			CA, %	Valid Tests, %	Errors, n (%) ^b,c^			CA ^c^, %
	R	I	S		VME	ME	mE			VME	ME	mE			VME	ME	mE	
All antibiotics	184	8	356	100	23 (12.5)	0	21 (3.8)	92.0	100	21 (11.4)	0	19 (3.5)	92.7	75.2	26 (18.1)	0	13 (3.2)	90.5
Ampicillin	24	NA	0	100	6	0	NA	75.0	100	4	0	NA	83.3	75.0	4	0	NA	77.8
Ampicillin/sulbactam	13	NA	11	100	4	0	NA	83.3	100	4	0	NA	83.3	75.0	3	0	NA	83.3
Piperacillin	15	0	9	100	1	0	1	91.7	100	1	0	1	91.7	75.0	2	0	0	88.9
Piperacillin/tazobactam	9	0	15	100	0	0	2	91.7	100	2	0	1	87.5	75.0	2	0	1	83.3
Cefuroxime ^d^	11	NA	3	100	0	0	NA	100	100	0	0	NA	100	78.6	1	0	NA	90.9
Cefotaxime	14	0	10	100	0	0	0	100	100	0	0	0	100	75.0	0	0	0	100
Ceftazidime	12	2	10	100	0	0	3	87.5	100	0	0	2	91.7	75.0	0	0	2	88.9
Ceftazidime/avibactam	1	NA	23	100	0	0	NA	100.0	100	0	0	NA	100	75.0	0	0	NA	100
Cefepime	11	1	12	100	2	0	5	70.8	100	1	0	5	75.0	75.0	1	0	3	77.8
Ceftolozane/tazobactam	9	NA	15	100	1	0	NA	95.8	100	1	0	NA	95.8	75.0	1	0	NA	94.4
Ertapenem	5	NA	19	100	0	0	NA	100	100	1	0	NA	95.8	75.0	1	0	NA	94.4
Imipenem	3	2	19	100	3	0	2	79.2	100	3	0	2	79.2	75.0	2	0	2	77.8
Meropenem	5	0	19	100	0	0	6	75.0	100	0	0	5	79.2	75.0	0	0	2	88.9
Aztreonam	13	1	10	100	1	0	1	91.7	100	0	0	3	87.5	75.0	0	0	2	88.9
Ciprofloxacin	6	2	16	100	0	0	0	100	100	0	0	0	100	75.0	1	0	1	88.9
Levofloxacin	6	0	18	100	0	0	0	100	100	0	0	0	100	75.0	1	0	0	94.4
Moxifloxacin	9	NA	15	100	1	0	NA	95.8	100	0	0	NA	100	75.0	0	0	NA	100
Gentamicin	1	0	23	100	0	0	0	100	100	0	0	0	100	75.0	0	0	0	100
Tobramycin	1	0	23	100	0	0	0	100	100	0	0	0	100	75.0	0	0	0	100
Amikacin	2	0	22	100	1	0	0	95.8	100	1	0	0	95.8	75.0	1	0	0	94.4
Trimethoprim/sulfamethoxazole	8	0	16	100	0	0	1	95.8	100	0	0	0	100	75.0	2	0	0	88.9
Colistin	3	NA	21	100	2	0	NA	91.7	100	2	0	NA	91.7	75.0	3	0	NA	83.3
Fosfomycin	3	NA	21	100	1	0	NA	95.8	100	1	0	NA	95.8	75.0	1	0	NA	94.4
Tigecycline ^e^	0	NA	6	100	0	0	NA	100	100	0	0	NA	100	83.3	0	0	NA	100

^a^ Number of isolate-antibiotic combinations that were categorized as resistant (R), susceptible, increased exposure (I) or susceptible, standard dosing regimen (S), according to the standard method. ^b^ The rates of very major error (VME, the number of false S results with DOT-MGA divided by the number of R results with standard method), major error (ME, the number of false R results with DOT-MGA divided by the number of S results with standard method) and minor error (mE, the number of false categorizations involving I category divided by the total number of tests) and categorical agreement (CA) are reported as required by ISO [20] and FDA [21]. ^c^ Calculated for valid tests (*n* = 412, all isolate-antibiotic combinations) in the 18-h setup. Number of isolate-antibiotic combinations that were categorized as R, I or S, according to the standard method, among valid tests was 144, 5 and 263, respectively. ^d^ Cefuroxime was only evaluated for *E. coli*, *Klebsiella* spp. (except *K. aerogenes*), *Raoultella* spp. and *P. mirabilis*, for which EUCAST breakpoints were available. ^e^ Tigecycline was only evaluated for *E. coli* and *C. koseri*, for which EUCAST breakpoints were available. NA, not applicable (no I category available from EUCAST).

## Data Availability

Data are contained within the article or Supplementary Material.

## Supplementary Materials

The following is available online at https://www.mdpi.com/article/10.3390/diagnostics11101803/s1, Table S1: panel for simultaneous DOT-MGA testing of multiple antibiotics vs. *Enterobacterales.*
