# Supplementary material for: Rapid Simultaneous Testing of Multiple Antibiotics by the MALDI-TOF MS Direct-on-Target Microdroplet Growth Assay
Source: Diagnostics (Basel). 2021 Sep 29;11(10):1803. doi: 10.3390/diagnostics11101803 (PMC8534412; doi:10.3390/diagnostics11101803)
Supplement: Supplementary file 1 [file diagnostics-11-01803-s001.zip › diagnostics-1390270-supplementary.pdf]

Rapid simultaneous testing of multiple antibiotics by the MALDI-TOF MS direct-on-target microdroplet growth assay

Evgeny A. Idelevich, Ilka D. Nix, Janika A. Busch, Katrin Sparbier, Oliver Drews, Markus Kostrzewa, Karsten Becker

Supplementary information

Table S1. Panel for simultaneous DOT-MGA testing of multiple antibiotics vs. *Enterobacterales*.

**Antimicrobial**	**Breakpoint concentrations**[a]
Amikacin	8 mg/L, 16 mg/L
Ampicillin	8 mg/L
Ampicillin/sulbactam	8/4 mg/L
Aztreonam	1 mg/L, 4 mg/L
Ceftazidime/avibactam	8/4 mg/L
Ceftazidime	1 mg/L, 4 mg/L
Cefepime	1 mg/L, 4 mg/L
Ciprofloxacin	0.25 mg/L, 0.5 mg/L
Colistin	2 mg/L
Ceftolozane/tazobactam	1/4 mg/L
Cefotaxime	1 mg/L, 2 mg/L
Cefuroxime	8 mg/L
Ertapenem	0.5 mg/L
Fosfomycin	32 mg/L
Gentamicin	2 mg/L, 4 mg/L
Imipenem	2 mg/L, 4 mg/L
Levofloxacin	0.5 mg/L, 1 mg/L
Meropenem	2 mg/L, 8 mg/L
Moxifloxacin	0.25 mg/L
Piperacillin	8 mg/L, 16 mg/L
Piperacillin/tazobactam	8/4 mg/L, 16/4 mg/L
Trimethoprim/sulfamethoxazole	2/38 mg/L, 4/76 mg/L
Tigecycline	0.5 mg/L
Tobramycin	2 mg/L, 4 mg/L

[a] Breakpoints according to the EUCAST breakpoint tables (version 9.0).
